# Supplementary material for: Genome-wide association studies reveal novel loci associated with pyrethroid and organophosphate resistance in Anopheles gambiae s.l
Source: bioRxiv. 2023 Jan 14:2023.01.13.523889. Preprint. [Version 1] doi: 10.1101/2023.01.13.523889 (PMC9882144; doi:10.1101/2023.01.13.523889)
Supplement: Supplement 5 [file media-5.zip › Supplementary_Data_S2.html]

 
 
 
   
   
   
   window_Fst_haplotype_summary 
   
   
     
   
 
 
 F ST  windows of interest 
 For each sample set, we first provide a summary plot of
F ST  bewteen resistant and susceptible samples across the
genome, with F ST  shown in red and the results of the 200
randomisations shown behind in grey. Windows identified as peaks are
highlighted by points, colour-coded by whether they are significantly
higher than expected based on the simulations (green) or not (purple).
For each significant window (green points), we then provide four
additional figures. The first shows the significant ( P  <
0.01) SNPs found in the region of that window, and their -log10(Pvalue)
of association with phenotype. Red points indicate non-synonymous SNPs,
blue points indicate all other SNPs. Point shape indicates whether the
mutant allele at that SNP is associated with increased (circle) or
decreased (triangle) resistance. Dark points in the centre of the plot
show SNPs within the significant window, light points on the sides show
SNPs in region 10,000 bp either side of the window. The second plot
shows the hierarchical cluster of haplotypes in the window, with
brackets indicating large haplotype clusters. The third plot shows, each
haplotype cluster, indicating the number of haplotypes comprising the
cluster (n), the  P  value of association of the cluster with
resistance phenotype, and the SNPs that differentiate that cluster from
other haplotypes in the region, colour-coded by type (red =
non-synonymous, yellow = synonymous, blue = non-coding). The final
figure shows the contingency tables of phenotype association for each
haplotype cluster. 
  Legend  
 Avrankou_ coluzzii _Delta  
 Baguida_ gambiae _Delta  
 Korle-Bu_ coluzzii _Delta  
 Madina_ gambiae _Delta  
 Obuasi_ gambiae _Delta  
 Baguida_ gambiae _PM  
 Korle-Bu_ coluzzii _PM  
 Madina_ gambiae _PM  
 Obuasi_ gambiae _PM  
 
 Plot legend 
   
   
 
 Avrankou_ coluzzii _Delta 
   
   
  Avrankou_coluzzii_Delta_2R:45413683-45433265  
 Avrankou_coluzzii_Delta_X:8745589-8778998  
 Avrankou_coluzzii_Delta_2R_45413683-45433265 
   
   
   
   
   
   
   
 Avrankou_coluzzii_Delta_X_8745589-8778998 
   
   
   
   
   
   
   
 Contingency table for
cluster_1: 
 
 
 
 
 
 
 
 
 
     phenotype     
     wt     
     het     
     hom     
 
 
 
 
 alive 
 11 
 22 
 12 
 
 
 dead 
 14 
 16 
 4 
 
 
 
   
 
 Baguida_ gambiae _Delta 
   
   
  Baguida_gambiae_Delta_2R:33967209-33981960  
 Baguida_gambiae_Delta_3L:3059554-3112922  
 Baguida_gambiae_Delta_2R_33967209-33981960 
   
   
   
   
   
   
   
 Contingency table for
cluster_1: 
 
 
 
 
 
 
 
 
 
     phenotype     
     wt     
     het     
     hom     
 
 
 
 
 alive 
 31 
 21 
 2 
 
 
 dead 
 31 
 3 
 0 
 
 
 
   
 Baguida_gambiae_Delta_3L_3059554-3112922 
   
   
   
   
   
   
   
 Contingency table for
cluster_1: 
 
 
 
 
 
 
 
 
 
     phenotype     
     wt     
     het     
     hom     
 
 
 
 
 alive 
 37 
 8 
 9 
 
 
 dead 
 11 
 21 
 2 
 
 
 
   
 Contingency table for
cluster_2: 
 
 
 
 
 
 
 
 
 
     phenotype     
     wt     
     het     
     hom     
 
 
 
 
 alive 
 39 
 14 
 1 
 
 
 dead 
 28 
 6 
 0 
 
 
 
   
 
 Korle-Bu_ coluzzii _Delta 
   
   
  Korle-Bu_coluzzii_Delta_2R:28145243-28168331  
 Korle-Bu_coluzzii_Delta_2R:39682306-39698081  
 Korle-Bu_coluzzii_Delta_3R:50971406-51021318  
 Korle-Bu_coluzzii_Delta_X:14653714-14668824  
 Korle-Bu_coluzzii_Delta_X:14668824-14684264  
 Korle-Bu_coluzzii_Delta_X:15670884-15702974  
 Korle-Bu_coluzzii_Delta_X:15702974-15756306  
 Korle-Bu_coluzzii_Delta_2R_28145243-28168331 
   
   
   
   
   
   
   
 Contingency table for
cluster_1: 
 
 
 
 
 
 
 
 
 
     phenotype     
     wt     
     het     
     hom     
 
 
 
 
 alive 
 52 
 7 
 0 
 
 
 dead 
 52 
 30 
 1 
 
 
 
   
 Contingency table for
cluster_2: 
 
 
 
 
 
 
 
 
 
     phenotype     
     wt     
     het     
     hom     
 
 
 
 
 alive 
 46 
 12 
 1 
 
 
 dead 
 69 
 13 
 1 
 
 
 
   
 Contingency table for
cluster_3: 
 
 
 
 
 
 
 
 
 
     phenotype     
     wt     
     het     
     hom     
 
 
 
 
 alive 
 50 
 9 
 0 
 
 
 dead 
 67 
 16 
 0 
 
 
 
   
 Contingency table for
cluster_4: 
 
 
 
 
 
 
 
 
 
     phenotype     
     wt     
     het     
     hom     
 
 
 
 
 alive 
 48 
 11 
 0 
 
 
 dead 
 72 
 11 
 0 
 
 
 
   
 Korle-Bu_coluzzii_Delta_2R_39682306-39698081 
   
   
   
   
   
   
   
 Contingency table for
cluster_1: 
 
 
 
 
 
 
 
 
 
     phenotype     
     wt     
     het     
     hom     
 
 
 
 
 alive 
 47 
 12 
 0 
 
 
 dead 
 65 
 16 
 2 
 
 
 
   
 Contingency table for
cluster_2: 
 
 
 
 
 
 
 
 
 
     phenotype     
     wt     
     het     
     hom     
 
 
 
 
 alive 
 42 
 16 
 1 
 
 
 dead 
 77 
 6 
 0 
 
 
 
   
 Korle-Bu_coluzzii_Delta_3R_50971406-51021318 
   
   
   
   
   
   
   
 Contingency table for
cluster_1: 
 
 
 
 
 
 
 
 
 
     phenotype     
     wt     
     het     
     hom     
 
 
 
 
 alive 
 44 
 13 
 2 
 
 
 dead 
 53 
 26 
 4 
 
 
 
   
 Contingency table for
cluster_2: 
 
 
 
 
 
 
 
 
 
     phenotype     
     wt     
     het     
     hom     
 
 
 
 
 alive 
 45 
 14 
 0 
 
 
 dead 
 75 
 7 
 1 
 
 
 
   
 Contingency table for
cluster_3: 
 
 
 
 
 
 
 
 
 
     phenotype     
     wt     
     het     
     hom     
 
 
 
 
 alive 
 49 
 10 
 0 
 
 
 dead 
 72 
 10 
 1 
 
 
 
   
 Korle-Bu_coluzzii_Delta_X_14653714-14668824 
   
   
   
   
   
   
   
 Contingency table for
cluster_1: 
 
 
 
 
 
 
 
 
 
     phenotype     
     wt     
     het     
     hom     
 
 
 
 
 alive 
 30 
 26 
 3 
 
 
 dead 
 58 
 24 
 1 
 
 
 
   
 Contingency table for
cluster_2: 
 
 
 
 
 
 
 
 
 
     phenotype     
     wt     
     het     
     hom     
 
 
 
 
 alive 
 49 
 9 
 1 
 
 
 dead 
 69 
 12 
 2 
 
 
 
   
 Contingency table for
cluster_3: 
 
 
 
 
 
 
 
 
 
     phenotype     
     wt     
     het     
     hom     
 
 
 
 
 alive 
 51 
 8 
 0 
 
 
 dead 
 70 
 13 
 0 
 
 
 
   
 Korle-Bu_coluzzii_Delta_X_14668824-14684264 
   
   
   
   
   
   
   
 Contingency table for
cluster_1: 
 
 
 
 
 
 
 
 
 
     phenotype     
     wt     
     het     
     hom     
 
 
 
 
 alive 
 31 
 25 
 3 
 
 
 dead 
 58 
 24 
 1 
 
 
 
   
 Contingency table for
cluster_2: 
 
 
 
 
 
 
 
 
 
     phenotype     
     wt     
     het     
     hom     
 
 
 
 
 alive 
 47 
 11 
 1 
 
 
 dead 
 69 
 12 
 2 
 
 
 
   
 Contingency table for
cluster_3: 
 
 
 
 
 
 
 
 
 
     phenotype     
     wt     
     het     
     hom     
 
 
 
 
 alive 
 51 
 8 
 0 
 
 
 dead 
 70 
 13 
 0 
 
 
 
   
 Korle-Bu_coluzzii_Delta_X_15670884-15702974 
   
   
   
   
   
   
   
 Contingency table for
cluster_1: 
 
 
 
 
 
 
 
 
 
     phenotype     
     wt     
     het     
     hom     
 
 
 
 
 alive 
 13 
 32 
 14 
 
 
 dead 
 37 
 35 
 11 
 
 
 
   
 Contingency table for
cluster_2: 
 
 
 
 
 
 
 
 
 
     phenotype     
     wt     
     het     
     hom     
 
 
 
 
 alive 
 52 
 7 
 0 
 
 
 dead 
 69 
 14 
 0 
 
 
 
   
 Contingency table for
cluster_3: 
 
 
 
 
 
 
 
 
 
     phenotype     
     wt     
     het     
     hom     
 
 
 
 
 alive 
 53 
 6 
 0 
 
 
 dead 
 68 
 15 
 0 
 
 
 
   
 Korle-Bu_coluzzii_Delta_X_15702974-15756306 
   
   
   
   
   
   
   
 Contingency table for
cluster_1: 
 
 
 
 
 
 
 
 
 
     phenotype     
     wt     
     het     
     hom     
 
 
 
 
 alive 
 12 
 38 
 9 
 
 
 dead 
 38 
 35 
 10 
 
 
 
   
 Contingency table for
cluster_2: 
 
 
 
 
 
 
 
 
 
     phenotype     
     wt     
     het     
     hom     
 
 
 
 
 alive 
 52 
 7 
 0 
 
 
 dead 
 68 
 14 
 1 
 
 
 
   
 Contingency table for
cluster_3: 
 
 
 
 
 
 
 
 
 
     phenotype     
     wt     
     het     
     hom     
 
 
 
 
 alive 
 52 
 7 
 0 
 
 
 dead 
 69 
 14 
 0 
 
 
 
   
 
 Madina_ gambiae _Delta 
   
   
  Madina_gambiae_Delta_2R:28401363-28416200  
 Madina_gambiae_Delta_2R:28454838-28474602  
 Madina_gambiae_Delta_2R_28401363-28416200 
   
   
   
   
   
   
   
 Contingency table for
cluster_1: 
 
 
 
 
 
 
 
 
 
     phenotype     
     wt     
     het     
     hom     
 
 
 
 
 alive 
 13 
 33 
 18 
 
 
 dead 
 14 
 21 
 2 
 
 
 
   
 Madina_gambiae_Delta_2R_28454838-28474602 
   
   
   
   
   
   
   
 Contingency table for
cluster_1: 
 
 
 
 
 
 
 
 
 
     phenotype     
     wt     
     het     
     hom     
 
 
 
 
 alive 
 8 
 33 
 23 
 
 
 dead 
 12 
 20 
 5 
 
 
 
   
 Contingency table for
cluster_2: 
 
 
 
 
 
 
 
 
 
     phenotype     
     wt     
     het     
     hom     
 
 
 
 
 alive 
 50 
 13 
 1 
 
 
 dead 
 26 
 10 
 1 
 
 
 
   
 
 Obuasi_ gambiae _Delta 
   
   
  Obuasi_gambiae_Delta_2R:28250033-28262588  
 Obuasi_gambiae_Delta_2R:28262588-28274893  
 Obuasi_gambiae_Delta_2R:28274893-28289873  
 Obuasi_gambiae_Delta_2R:28289873-28306606  
 Obuasi_gambiae_Delta_2R:28306605-28322681  
 Obuasi_gambiae_Delta_2R:28322681-28337951  
 Obuasi_gambiae_Delta_2R:28337951-28354693  
 Obuasi_gambiae_Delta_2R:28354692-28369784  
 Obuasi_gambiae_Delta_2R:28369784-28391434  
 Obuasi_gambiae_Delta_2R:28391433-28405240  
 Obuasi_gambiae_Delta_2R:28405239-28421116  
 Obuasi_gambiae_Delta_2R:28421116-28439220  
 Obuasi_gambiae_Delta_2R:28439219-28456407  
 Obuasi_gambiae_Delta_2R:28456407-28475225  
 Obuasi_gambiae_Delta_2R:28475225-28496115  
 Obuasi_gambiae_Delta_2R:28496114-28515482  
 Obuasi_gambiae_Delta_2R:28515481-28531112  
 Obuasi_gambiae_Delta_2R_28250033-28262588 
   
   
   
   
   
   
   
 Contingency table for
cluster_1: 
 
 
 
 
 
 
 
 
 
     phenotype     
     wt     
     het     
     hom     
 
 
 
 
 alive 
 11 
 16 
 0 
 
 
 dead 
 39 
 12 
 0 
 
 
 
   
 Contingency table for
cluster_2: 
 
 
 
 
 
 
 
 
 
     phenotype     
     wt     
     het     
     hom     
 
 
 
 
 alive 
 23 
 4 
 0 
 
 
 dead 
 33 
 16 
 2 
 
 
 
   
 Obuasi_gambiae_Delta_2R_28262588-28274893 
   
   
   
   
   
   
   
 Contingency table for
cluster_1: 
 
 
 
 
 
 
 
 
 
     phenotype     
     wt     
     het     
     hom     
 
 
 
 
 alive 
 10 
 15 
 2 
 
 
 dead 
 39 
 12 
 0 
 
 
 
   
 Contingency table for
cluster_2: 
 
 
 
 
 
 
 
 
 
     phenotype     
     wt     
     het     
     hom     
 
 
 
 
 alive 
 23 
 4 
 0 
 
 
 dead 
 33 
 16 
 2 
 
 
 
   
 Obuasi_gambiae_Delta_2R_28274893-28289873 
   
   
   
   
   
   
   
 Contingency table for
cluster_1: 
 
 
 
 
 
 
 
 
 
     phenotype     
     wt     
     het     
     hom     
 
 
 
 
 alive 
 10 
 14 
 3 
 
 
 dead 
 39 
 12 
 0 
 
 
 
   
 Contingency table for
cluster_2: 
 
 
 
 
 
 
 
 
 
     phenotype     
     wt     
     het     
     hom     
 
 
 
 
 alive 
 23 
 4 
 0 
 
 
 dead 
 33 
 16 
 2 
 
 
 
   
 Obuasi_gambiae_Delta_2R_28289873-28306606 
   
   
   
   
   
   
   
 Contingency table for
cluster_1: 
 
 
 
 
 
 
 
 
 
     phenotype     
     wt     
     het     
     hom     
 
 
 
 
 alive 
 9 
 14 
 4 
 
 
 dead 
 39 
 12 
 0 
 
 
 
   
 Contingency table for
cluster_2: 
 
 
 
 
 
 
 
 
 
     phenotype     
     wt     
     het     
     hom     
 
 
 
 
 alive 
 23 
 4 
 0 
 
 
 dead 
 33 
 17 
 1 
 
 
 
   
 Obuasi_gambiae_Delta_2R_28306605-28322681 
   
   
   
   
   
   
   
 Contingency table for
cluster_1: 
 
 
 
 
 
 
 
 
 
     phenotype     
     wt     
     het     
     hom     
 
 
 
 
 alive 
 10 
 14 
 3 
 
 
 dead 
 38 
 13 
 0 
 
 
 
   
 Contingency table for
cluster_2: 
 
 
 
 
 
 
 
 
 
     phenotype     
     wt     
     het     
     hom     
 
 
 
 
 alive 
 23 
 4 
 0 
 
 
 dead 
 33 
 17 
 1 
 
 
 
   
 Obuasi_gambiae_Delta_2R_28322681-28337951 
   
   
   
   
   
   
   
 Contingency table for
cluster_1: 
 
 
 
 
 
 
 
 
 
     phenotype     
     wt     
     het     
     hom     
 
 
 
 
 alive 
 9 
 14 
 4 
 
 
 dead 
 37 
 14 
 0 
 
 
 
   
 Contingency table for
cluster_2: 
 
 
 
 
 
 
 
 
 
     phenotype     
     wt     
     het     
     hom     
 
 
 
 
 alive 
 23 
 4 
 0 
 
 
 dead 
 34 
 16 
 1 
 
 
 
   
 Contingency table for
cluster_3: 
 
 
 
 
 
 
 
 
 
     phenotype     
     wt     
     het     
     hom     
 
 
 
 
 alive 
 24 
 3 
 0 
 
 
 dead 
 34 
 16 
 1 
 
 
 
   
 Obuasi_gambiae_Delta_2R_28337951-28354693 
   
   
   
   
   
   
   
 Contingency table for
cluster_1: 
 
 
 
 
 
 
 
 
 
     phenotype     
     wt     
     het     
     hom     
 
 
 
 
 alive 
 9 
 14 
 4 
 
 
 dead 
 36 
 15 
 0 
 
 
 
   
 Contingency table for
cluster_2: 
 
 
 
 
 
 
 
 
 
     phenotype     
     wt     
     het     
     hom     
 
 
 
 
 alive 
 24 
 3 
 0 
 
 
 dead 
 33 
 17 
 1 
 
 
 
   
 Contingency table for
cluster_3: 
 
 
 
 
 
 
 
 
 
     phenotype     
     wt     
     het     
     hom     
 
 
 
 
 alive 
 22 
 5 
 0 
 
 
 dead 
 36 
 14 
 1 
 
 
 
   
 Obuasi_gambiae_Delta_2R_28354692-28369784 
   
   
   
   
   
   
   
 Contingency table for
cluster_1: 
 
 
 
 
 
 
 
 
 
     phenotype     
     wt     
     het     
     hom     
 
 
 
 
 alive 
 8 
 15 
 4 
 
 
 dead 
 35 
 16 
 0 
 
 
 
   
 Contingency table for
cluster_2: 
 
 
 
 
 
 
 
 
 
     phenotype     
     wt     
     het     
     hom     
 
 
 
 
 alive 
 24 
 3 
 0 
 
 
 dead 
 32 
 17 
 2 
 
 
 
   
 Contingency table for
cluster_3: 
 
 
 
 
 
 
 
 
 
     phenotype     
     wt     
     het     
     hom     
 
 
 
 
 alive 
 21 
 6 
 0 
 
 
 dead 
 36 
 14 
 1 
 
 
 
   
 Obuasi_gambiae_Delta_2R_28369784-28391434 
   
   
   
   
   
   
   
 Contingency table for
cluster_1: 
 
 
 
 
 
 
 
 
 
     phenotype     
     wt     
     het     
     hom     
 
 
 
 
 alive 
 8 
 15 
 4 
 
 
 dead 
 35 
 15 
 1 
 
 
 
   
 Contingency table for
cluster_2: 
 
 
 
 
 
 
 
 
 
     phenotype     
     wt     
     het     
     hom     
 
 
 
 
 alive 
 24 
 3 
 0 
 
 
 dead 
 32 
 17 
 2 
 
 
 
   
 Contingency table for
cluster_3: 
 
 
 
 
 
 
 
 
 
     phenotype     
     wt     
     het     
     hom     
 
 
 
 
 alive 
 21 
 6 
 0 
 
 
 dead 
 35 
 15 
 1 
 
 
 
   
 Obuasi_gambiae_Delta_2R_28391433-28405240 
   
   
   
   
   
   
   
 Contingency table for
cluster_1: 
 
 
 
 
 
 
 
 
 
     phenotype     
     wt     
     het     
     hom     
 
 
 
 
 alive 
 7 
 16 
 4 
 
 
 dead 
 34 
 15 
 2 
 
 
 
   
 Contingency table for
cluster_2: 
 
 
 
 
 
 
 
 
 
     phenotype     
     wt     
     het     
     hom     
 
 
 
 
 alive 
 24 
 3 
 0 
 
 
 dead 
 32 
 17 
 2 
 
 
 
   
 Contingency table for
cluster_3: 
 
 
 
 
 
 
 
 
 
     phenotype     
     wt     
     het     
     hom     
 
 
 
 
 alive 
 21 
 6 
 0 
 
 
 dead 
 35 
 15 
 1 
 
 
 
   
 Obuasi_gambiae_Delta_2R_28405239-28421116 
   
   
   
   
   
   
   
 Contingency table for
cluster_1: 
 
 
 
 
 
 
 
 
 
     phenotype     
     wt     
     het     
     hom     
 
 
 
 
 alive 
 9 
 11 
 7 
 
 
 dead 
 32 
 17 
 2 
 
 
 
   
 Contingency table for
cluster_2: 
 
 
 
 
 
 
 
 
 
     phenotype     
     wt     
     het     
     hom     
 
 
 
 
 alive 
 21 
 6 
 0 
 
 
 dead 
 35 
 15 
 1 
 
 
 
   
 Contingency table for
cluster_3: 
 
 
 
 
 
 
 
 
 
     phenotype     
     wt     
     het     
     hom     
 
 
 
 
 alive 
 24 
 3 
 0 
 
 
 dead 
 33 
 16 
 2 
 
 
 
   
 Obuasi_gambiae_Delta_2R_28421116-28439220 
   
   
   
   
   
   
   
 Contingency table for
cluster_1: 
 
 
 
 
 
 
 
 
 
     phenotype     
     wt     
     het     
     hom     
 
 
 
 
 alive 
 7 
 13 
 7 
 
 
 dead 
 29 
 18 
 4 
 
 
 
   
 Contingency table for
cluster_2: 
 
 
 
 
 
 
 
 
 
     phenotype     
     wt     
     het     
     hom     
 
 
 
 
 alive 
 24 
 3 
 0 
 
 
 dead 
 33 
 16 
 2 
 
 
 
   
 Contingency table for
cluster_3: 
 
 
 
 
 
 
 
 
 
     phenotype     
     wt     
     het     
     hom     
 
 
 
 
 alive 
 21 
 6 
 0 
 
 
 dead 
 36 
 14 
 1 
 
 
 
   
 Obuasi_gambiae_Delta_2R_28439219-28456407 
   
   
   
   
   
   
   
 Contingency table for
cluster_1: 
 
 
 
 
 
 
 
 
 
     phenotype     
     wt     
     het     
     hom     
 
 
 
 
 alive 
 4 
 14 
 9 
 
 
 dead 
 27 
 17 
 7 
 
 
 
   
 Contingency table for
cluster_2: 
 
 
 
 
 
 
 
 
 
     phenotype     
     wt     
     het     
     hom     
 
 
 
 
 alive 
 23 
 4 
 0 
 
 
 dead 
 32 
 17 
 2 
 
 
 
   
 Contingency table for
cluster_3: 
 
 
 
 
 
 
 
 
 
     phenotype     
     wt     
     het     
     hom     
 
 
 
 
 alive 
 20 
 7 
 0 
 
 
 dead 
 36 
 14 
 1 
 
 
 
   
 Obuasi_gambiae_Delta_2R_28456407-28475225 
   
   
   
   
   
   
   
 Contingency table for
cluster_1: 
 
 
 
 
 
 
 
 
 
     phenotype     
     wt     
     het     
     hom     
 
 
 
 
 alive 
 3 
 15 
 9 
 
 
 dead 
 25 
 19 
 7 
 
 
 
   
 Contingency table for
cluster_2: 
 
 
 
 
 
 
 
 
 
     phenotype     
     wt     
     het     
     hom     
 
 
 
 
 alive 
 23 
 4 
 0 
 
 
 dead 
 32 
 17 
 2 
 
 
 
   
 Contingency table for
cluster_3: 
 
 
 
 
 
 
 
 
 
     phenotype     
     wt     
     het     
     hom     
 
 
 
 
 alive 
 20 
 7 
 0 
 
 
 dead 
 38 
 12 
 1 
 
 
 
   
 Obuasi_gambiae_Delta_2R_28475225-28496115 
   
   
   
   
   
   
   
 Contingency table for
cluster_1: 
 
 
 
 
 
 
 
 
 
     phenotype     
     wt     
     het     
     hom     
 
 
 
 
 alive 
 7 
 12 
 8 
 
 
 dead 
 28 
 18 
 5 
 
 
 
   
 Contingency table for
cluster_2: 
 
 
 
 
 
 
 
 
 
     phenotype     
     wt     
     het     
     hom     
 
 
 
 
 alive 
 19 
 8 
 0 
 
 
 dead 
 39 
 11 
 1 
 
 
 
   
 Obuasi_gambiae_Delta_2R_28496114-28515482 
   
   
   
   
   
   
   
 Contingency table for
cluster_1: 
 
 
 
 
 
 
 
 
 
     phenotype     
     wt     
     het     
     hom     
 
 
 
 
 alive 
 11 
 9 
 7 
 
 
 dead 
 30 
 16 
 5 
 
 
 
   
 Contingency table for
cluster_2: 
 
 
 
 
 
 
 
 
 
     phenotype     
     wt     
     het     
     hom     
 
 
 
 
 alive 
 17 
 10 
 0 
 
 
 dead 
 38 
 12 
 1 
 
 
 
   
 Obuasi_gambiae_Delta_2R_28515481-28531112 
   
   
   
   
   
   
   
 Contingency table for
cluster_1: 
 
 
 
 
 
 
 
 
 
     phenotype     
     wt     
     het     
     hom     
 
 
 
 
 alive 
 13 
 11 
 3 
 
 
 dead 
 35 
 12 
 4 
 
 
 
   
 Contingency table for
cluster_2: 
 
 
 
 
 
 
 
 
 
     phenotype     
     wt     
     het     
     hom     
 
 
 
 
 alive 
 14 
 12 
 1 
 
 
 dead 
 38 
 12 
 1 
 
 
 
   
 
 Baguida_ gambiae _PM 
   
   
  Baguida_gambiae_PM_X:15411876-15495650  
 Baguida_gambiae_PM_X:9165646-9222299  
 Baguida_gambiae_PM_X:9222298-9273975  
 Baguida_gambiae_PM_X:9273975-9317640  
 Baguida_gambiae_PM_X:9317639-9346605  
 Baguida_gambiae_PM_X_15411876-15495650 
   
   
   
   
   
   
   
 Contingency table for
cluster_1: 
 
 
 
 
 
 
 
 
 
     phenotype     
     wt     
     het     
     hom     
 
 
 
 
 alive 
 17 
 14 
 4 
 
 
 dead 
 13 
 12 
 5 
 
 
 
   
 Contingency table for
cluster_2: 
 
 
 
 
 
 
 
 
 
     phenotype     
     wt     
     het     
     hom     
 
 
 
 
 alive 
 24 
 11 
 0 
 
 
 dead 
 12 
 16 
 2 
 
 
 
   
 Contingency table for
cluster_3: 
 
 
 
 
 
 
 
 
 
     phenotype     
     wt     
     het     
     hom     
 
 
 
 
 alive 
 20 
 11 
 4 
 
 
 dead 
 24 
 5 
 1 
 
 
 
   
 Baguida_gambiae_PM_X_9165646-9222299 
   
   
   
   
   
   
   
 Contingency table for
cluster_1: 
 
 
 
 
 
 
 
 
 
     phenotype     
     wt     
     het     
     hom     
 
 
 
 
 alive 
 6 
 13 
 16 
 
 
 dead 
 9 
 13 
 8 
 
 
 
   
 Contingency table for
cluster_2: 
 
 
 
 
 
 
 
 
 
     phenotype     
     wt     
     het     
     hom     
 
 
 
 
 alive 
 24 
 10 
 1 
 
 
 dead 
 10 
 14 
 6 
 
 
 
   
 Baguida_gambiae_PM_X_9222298-9273975 
   
   
   
   
   
   
   
 Contingency table for
cluster_1: 
 
 
 
 
 
 
 
 
 
     phenotype     
     wt     
     het     
     hom     
 
 
 
 
 alive 
 16 
 13 
 6 
 
 
 dead 
 13 
 15 
 2 
 
 
 
   
 Contingency table for
cluster_2: 
 
 
 
 
 
 
 
 
 
     phenotype     
     wt     
     het     
     hom     
 
 
 
 
 alive 
 24 
 10 
 1 
 
 
 dead 
 10 
 14 
 6 
 
 
 
   
 Baguida_gambiae_PM_X_9273975-9317640 
   
   
   
   
   
   
   
 Contingency table for
cluster_1: 
 
 
 
 
 
 
 
 
 
     phenotype     
     wt     
     het     
     hom     
 
 
 
 
 alive 
 24 
 10 
 1 
 
 
 dead 
 10 
 14 
 6 
 
 
 
   
 Contingency table for
cluster_2: 
 
 
 
 
 
 
 
 
 
     phenotype     
     wt     
     het     
     hom     
 
 
 
 
 alive 
 20 
 11 
 4 
 
 
 dead 
 19 
 11 
 0 
 
 
 
   
 Baguida_gambiae_PM_X_9317639-9346605 
   
   
   
   
   
   
   
 Contingency table for
cluster_1: 
 
 
 
 
 
 
 
 
 
     phenotype     
     wt     
     het     
     hom     
 
 
 
 
 alive 
 24 
 10 
 1 
 
 
 dead 
 11 
 14 
 5 
 
 
 
   
 Contingency table for
cluster_2: 
 
 
 
 
 
 
 
 
 
     phenotype     
     wt     
     het     
     hom     
 
 
 
 
 alive 
 19 
 14 
 2 
 
 
 dead 
 22 
 8 
 0 
 
 
 
   
 
 Korle-Bu_ coluzzii _PM 
   
   
  Korle-Bu_coluzzii_PM_2R:38097626-38111826  
 Korle-Bu_coluzzii_PM_2R:54371673-54397846  
 Korle-Bu_coluzzii_PM_3R:28482794-28505651  
 Korle-Bu_coluzzii_PM_3R:28505651-28533744  
 Korle-Bu_coluzzii_PM_3R:28563392-28595112  
 Korle-Bu_coluzzii_PM_3R:2929236-2946500  
 Korle-Bu_coluzzii_PM_2R_38097626-38111826 
   
   
   
   
   
   
   
 Korle-Bu_coluzzii_PM_2R_54371673-54397846 
   
   
   
   
   
   
   
 Contingency table for
cluster_1: 
 
 
 
 
 
 
 
 
 
     phenotype     
     wt     
     het     
     hom     
 
 
 
 
 alive 
 30 
 22 
 5 
 
 
 dead 
 34 
 9 
 5 
 
 
 
   
 Korle-Bu_coluzzii_PM_3R_28482794-28505651 
   
   
   
   
   
   
   
 Contingency table for
cluster_1: 
 
 
 
 
 
 
 
 
 
     phenotype     
     wt     
     het     
     hom     
 
 
 
 
 alive 
 34 
 20 
 3 
 
 
 dead 
 14 
 28 
 6 
 
 
 
   
 Contingency table for
cluster_2: 
 
 
 
 
 
 
 
 
 
     phenotype     
     wt     
     het     
     hom     
 
 
 
 
 alive 
 34 
 19 
 4 
 
 
 dead 
 40 
 8 
 0 
 
 
 
   
 Contingency table for
cluster_3: 
 
 
 
 
 
 
 
 
 
     phenotype     
     wt     
     het     
     hom     
 
 
 
 
 alive 
 39 
 17 
 1 
 
 
 dead 
 41 
 6 
 1 
 
 
 
   
 Korle-Bu_coluzzii_PM_3R_28505651-28533744 
   
   
   
   
   
   
   
 Contingency table for
cluster_1: 
 
 
 
 
 
 
 
 
 
     phenotype     
     wt     
     het     
     hom     
 
 
 
 
 alive 
 37 
 17 
 3 
 
 
 dead 
 15 
 29 
 4 
 
 
 
   
 Contingency table for
cluster_2: 
 
 
 
 
 
 
 
 
 
     phenotype     
     wt     
     het     
     hom     
 
 
 
 
 alive 
 26 
 24 
 7 
 
 
 dead 
 31 
 16 
 1 
 
 
 
   
 Contingency table for
cluster_3: 
 
 
 
 
 
 
 
 
 
     phenotype     
     wt     
     het     
     hom     
 
 
 
 
 alive 
 39 
 15 
 3 
 
 
 dead 
 40 
 7 
 1 
 
 
 
   
 Korle-Bu_coluzzii_PM_3R_28563392-28595112 
   
   
   
   
   
   
   
 Contingency table for
cluster_1: 
 
 
 
 
 
 
 
 
 
     phenotype     
     wt     
     het     
     hom     
 
 
 
 
 alive 
 20 
 27 
 10 
 
 
 dead 
 20 
 25 
 3 
 
 
 
   
 Contingency table for
cluster_2: 
 
 
 
 
 
 
 
 
 
     phenotype     
     wt     
     het     
     hom     
 
 
 
 
 alive 
 38 
 17 
 2 
 
 
 dead 
 18 
 25 
 5 
 
 
 
   
 Contingency table for
cluster_3: 
 
 
 
 
 
 
 
 
 
     phenotype     
     wt     
     het     
     hom     
 
 
 
 
 alive 
 38 
 16 
 3 
 
 
 dead 
 41 
 6 
 1 
 
 
 
   
 Korle-Bu_coluzzii_PM_3R_2929236-2946500 
   
   
   
   
   
   
   
 Contingency table for
cluster_1: 
 
 
 
 
 
 
 
 
 
     phenotype     
     wt     
     het     
     hom     
 
 
 
 
 alive 
 24 
 26 
 7 
 
 
 dead 
 7 
 27 
 14 
 
 
 
   
 
 Madina_ gambiae _PM 
   
   
  Madina_gambiae_PM_2R:54255612-54278008  
 Madina_gambiae_PM_2R_54255612-54278008 
   
   
   
   
   
   
   
 Contingency table for
cluster_1: 
 
 
 
 
 
 
 
 
 
     phenotype     
     wt     
     het     
     hom     
 
 
 
 
 alive 
 31 
 7 
 0 
 
 
 dead 
 14 
 12 
 1 
 
 
 
   
 
 Obuasi_ gambiae _PM 
   
   
  Obuasi_gambiae_PM_2R:3474360-3509400  
 Obuasi_gambiae_PM_3L:30477135-30487616  
 Obuasi_gambiae_PM_3L:30487615-30497759  
 Obuasi_gambiae_PM_3L:30497758-30506894  
 Obuasi_gambiae_PM_3L:30506893-30516797  
 Obuasi_gambiae_PM_3L:30534828-30545854  
 Obuasi_gambiae_PM_3L:30554459-30562573  
 Obuasi_gambiae_PM_3L:30562572-30578511  
 Obuasi_gambiae_PM_3L:30578511-30587941  
 Obuasi_gambiae_PM_3L:30587941-30597762  
 Obuasi_gambiae_PM_3L:30597761-30607138  
 Obuasi_gambiae_PM_3L:30607137-30617085  
 Obuasi_gambiae_PM_3L:30617085-30624950  
 Obuasi_gambiae_PM_3L:30624949-30635698  
 Obuasi_gambiae_PM_3L:30635698-30646602  
 Obuasi_gambiae_PM_3L:30646601-30657763  
 Obuasi_gambiae_PM_3L:30657762-30667355  
 Obuasi_gambiae_PM_3L:30667355-30678119  
 Obuasi_gambiae_PM_3L:30678119-30687769  
 Obuasi_gambiae_PM_3L:30687769-30697180  
 Obuasi_gambiae_PM_3R:48454456-48494640  
 Obuasi_gambiae_PM_2R_3474360-3509400 
   
   
   
   
   
   
   
 Contingency table for
cluster_1: 
 
 
 
 
 
 
 
 
 
     phenotype     
     wt     
     het     
     hom     
 
 
 
 
 alive 
 1 
 2 
 51 
 
 
 dead 
 1 
 16 
 33 
 
 
 
   
 Obuasi_gambiae_PM_3L_30477135-30487616 
   
   
   
   
   
   
   
 Contingency table for
cluster_1: 
 
 
 
 
 
 
 
 
 
     phenotype     
     wt     
     het     
     hom     
 
 
 
 
 alive 
 37 
 16 
 1 
 
 
 dead 
 17 
 28 
 5 
 
 
 
   
 Obuasi_gambiae_PM_3L_30487615-30497759 
   
   
   
   
   
   
   
 Contingency table for
cluster_1: 
 
 
 
 
 
 
 
 
 
     phenotype     
     wt     
     het     
     hom     
 
 
 
 
 alive 
 33 
 20 
 1 
 
 
 dead 
 14 
 29 
 7 
 
 
 
   
 Obuasi_gambiae_PM_3L_30497758-30506894 
   
   
   
   
   
   
   
 Contingency table for
cluster_1: 
 
 
 
 
 
 
 
 
 
     phenotype     
     wt     
     het     
     hom     
 
 
 
 
 alive 
 36 
 17 
 1 
 
 
 dead 
 13 
 31 
 6 
 
 
 
   
 Obuasi_gambiae_PM_3L_30506893-30516797 
   
   
   
   
   
   
   
 Contingency table for
cluster_1: 
 
 
 
 
 
 
 
 
 
     phenotype     
     wt     
     het     
     hom     
 
 
 
 
 alive 
 35 
 19 
 0 
 
 
 dead 
 15 
 29 
 6 
 
 
 
   
 Obuasi_gambiae_PM_3L_30534828-30545854 
   
   
   
   
   
   
   
 Contingency table for
cluster_1: 
 
 
 
 
 
 
 
 
 
     phenotype     
     wt     
     het     
     hom     
 
 
 
 
 alive 
 35 
 18 
 1 
 
 
 dead 
 15 
 31 
 4 
 
 
 
   
 Obuasi_gambiae_PM_3L_30554459-30562573 
   
   
   
   
   
   
   
 Contingency table for
cluster_1: 
 
 
 
 
 
 
 
 
 
     phenotype     
     wt     
     het     
     hom     
 
 
 
 
 alive 
 36 
 17 
 1 
 
 
 dead 
 16 
 29 
 5 
 
 
 
   
 Obuasi_gambiae_PM_3L_30562572-30578511 
   
   
   
   
   
   
   
 Contingency table for
cluster_1: 
 
 
 
 
 
 
 
 
 
     phenotype     
     wt     
     het     
     hom     
 
 
 
 
 alive 
 36 
 16 
 2 
 
 
 dead 
 16 
 29 
 5 
 
 
 
   
 Obuasi_gambiae_PM_3L_30578511-30587941 
   
   
   
   
   
   
   
 Contingency table for
cluster_1: 
 
 
 
 
 
 
 
 
 
     phenotype     
     wt     
     het     
     hom     
 
 
 
 
 alive 
 35 
 17 
 2 
 
 
 dead 
 15 
 28 
 7 
 
 
 
   
 Obuasi_gambiae_PM_3L_30587941-30597762 
   
   
   
   
   
   
   
 Contingency table for
cluster_1: 
 
 
 
 
 
 
 
 
 
     phenotype     
     wt     
     het     
     hom     
 
 
 
 
 alive 
 35 
 17 
 2 
 
 
 dead 
 15 
 28 
 7 
 
 
 
   
 Obuasi_gambiae_PM_3L_30597761-30607138 
   
   
   
   
   
   
   
 Contingency table for
cluster_1: 
 
 
 
 
 
 
 
 
 
     phenotype     
     wt     
     het     
     hom     
 
 
 
 
 alive 
 35 
 17 
 2 
 
 
 dead 
 15 
 28 
 7 
 
 
 
   
 Obuasi_gambiae_PM_3L_30607137-30617085 
   
   
   
   
   
   
   
 Contingency table for
cluster_1: 
 
 
 
 
 
 
 
 
 
     phenotype     
     wt     
     het     
     hom     
 
 
 
 
 alive 
 34 
 18 
 2 
 
 
 dead 
 15 
 27 
 8 
 
 
 
   
 Obuasi_gambiae_PM_3L_30617085-30624950 
   
   
   
   
   
   
   
 Contingency table for
cluster_1: 
 
 
 
 
 
 
 
 
 
     phenotype     
     wt     
     het     
     hom     
 
 
 
 
 alive 
 33 
 18 
 3 
 
 
 dead 
 15 
 25 
 10 
 
 
 
   
 Obuasi_gambiae_PM_3L_30624949-30635698 
   
   
   
   
   
   
   
 Contingency table for
cluster_1: 
 
 
 
 
 
 
 
 
 
     phenotype     
     wt     
     het     
     hom     
 
 
 
 
 alive 
 30 
 21 
 3 
 
 
 dead 
 10 
 28 
 12 
 
 
 
   
 Obuasi_gambiae_PM_3L_30635698-30646602 
   
   
   
   
   
   
   
 Contingency table for
cluster_1: 
 
 
 
 
 
 
 
 
 
     phenotype     
     wt     
     het     
     hom     
 
 
 
 
 alive 
 30 
 21 
 3 
 
 
 dead 
 10 
 28 
 12 
 
 
 
   
 Obuasi_gambiae_PM_3L_30646601-30657763 
   
   
   
   
   
   
   
 Contingency table for
cluster_1: 
 
 
 
 
 
 
 
 
 
     phenotype     
     wt     
     het     
     hom     
 
 
 
 
 alive 
 28 
 24 
 2 
 
 
 dead 
 11 
 29 
 10 
 
 
 
   
 Obuasi_gambiae_PM_3L_30657762-30667355 
   
   
   
   
   
   
   
 Contingency table for
cluster_1: 
 
 
 
 
 
 
 
 
 
     phenotype     
     wt     
     het     
     hom     
 
 
 
 
 alive 
 28 
 24 
 2 
 
 
 dead 
 11 
 30 
 9 
 
 
 
   
 Obuasi_gambiae_PM_3L_30667355-30678119 
   
   
   
   
   
   
   
 Contingency table for
cluster_1: 
 
 
 
 
 
 
 
 
 
     phenotype     
     wt     
     het     
     hom     
 
 
 
 
 alive 
 27 
 25 
 2 
 
 
 dead 
 10 
 31 
 9 
 
 
 
   
 Obuasi_gambiae_PM_3L_30678119-30687769 
   
   
   
   
   
   
   
 Contingency table for
cluster_1: 
 
 
 
 
 
 
 
 
 
     phenotype     
     wt     
     het     
     hom     
 
 
 
 
 alive 
 26 
 26 
 2 
 
 
 dead 
 11 
 29 
 10 
 
 
 
   
 Obuasi_gambiae_PM_3L_30687769-30697180 
   
   
   
   
   
   
   
 Contingency table for
cluster_1: 
 
 
 
 
 
 
 
 
 
     phenotype     
     wt     
     het     
     hom     
 
 
 
 
 alive 
 26 
 26 
 2 
 
 
 dead 
 9 
 30 
 11 
 
 
 
   
 Obuasi_gambiae_PM_3R_48454456-48494640 
   
   
   
   
   
   
   
 Contingency table for
cluster_1: 
 
 
 
 
 
 
 
 
 
     phenotype     
     wt     
     het     
     hom     
 
 
 
 
 alive 
 24 
 24 
 6 
 
 
 dead 
 39 
 9 
 2 
 
 
 
   
 Contingency table for
cluster_2: 
 
 
 
 
 
 
 
 
 
     phenotype     
     wt     
     het     
     hom     
 
 
 
 
 alive 
 43 
 10 
 1 
 
 
 dead 
 38 
 9 
 3 
 
 
 
   
 Contingency table for
cluster_3: 
 
 
 
 
 
 
 
 
 
     phenotype     
     wt     
     het     
     hom     
 
 
 
 
 alive 
 44 
 10 
 0 
 
 
 dead 
 39 
 10 
 1 
 
 
 
   
 
 
 
