# Supplementary material for: Genome-wide association studies reveal novel loci associated with pyrethroid and organophosphate resistance in Anopheles gambiae s.l
Source: bioRxiv. 2023 Jan 14:2023.01.13.523889. Preprint. [Version 1] doi: 10.1101/2023.01.13.523889 (PMC9882144; doi:10.1101/2023.01.13.523889)
Supplement: Supplement 7 [file media-7.zip › Supplementary_Data_S5.html]

 
 
 
   
   
   
   window_PBS_summary 
   
   
     
   
 
 
 PBS windows of interest 
 For each sample set, we first provide a summary plot of PBS across
the genome, with PBS shown in blue and the results of the 200
randomisations shown behind in grey. Windows identified as peaks are
highlighted by points, colour-coded by whether they are significantly
higher than expected based on the simulations (green) or not (purple).
For each significant window (green points), we then provide its own plot
showing the significant ( P  < 0.01) SNPs found in the region
of that window, and their -log10(Pvalue) of association with phenotype.
Red points indicate non-synonymous SNPs, blue points indicate all other
SNPs. Point shape indicates whether the mutant allele at that SNP is
associated with increased (circle) or decreased (triangle) resistance.
Dark points in the centre of the plot show SNPs within the significant
window, light points on the sides show SNPs in the region 10,000 bp
either side of the window. 
  Legend  
 Avrankou_ coluzzii _Delta  
 Baguida_ gambiae _Delta  
 Korle-Bu_ coluzzii _Delta  
 Madina_ gambiae _Delta  
 Obuasi_ gambiae _Delta  
 Baguida_ gambiae _PM  
 Korle-Bu_ coluzzii _PM  
 Madina_ gambiae _PM  
 Obuasi_ gambiae _PM  
 
 Plot legend 
   
   
 
 Avrankou_ coluzzii _Delta 
   
   
  Avrankou_coluzzii_Delta_2L:10075762  
 Avrankou_coluzzii_Delta_2R:36536888  
 Avrankou_coluzzii_Delta_2R:40059143  
 Avrankou_coluzzii_Delta_2R:50397087  
 Avrankou_coluzzii_Delta_2R:57936779  
 Avrankou_coluzzii_Delta_2R:58017231  
 Avrankou_coluzzii_Delta_3R:40583507  
 Avrankou_coluzzii_Delta_3R:42095383  
 Avrankou_coluzzii_Delta_3R:43486612  
 Avrankou_coluzzii_Delta_3R:43532647  
 Avrankou_coluzzii_Delta_X:16349957  
 Avrankou_coluzzii_Delta_2L_10075762 
   
   
   
 Avrankou_coluzzii_Delta_2R_36536888 
   
   
   
 Avrankou_coluzzii_Delta_2R_40059143 
   
   
   
 Avrankou_coluzzii_Delta_2R_50397087 
   
   
   
 Avrankou_coluzzii_Delta_2R_57936779 
   
   
   
 Avrankou_coluzzii_Delta_2R_58017231 
   
   
   
 Avrankou_coluzzii_Delta_3R_40583507 
   
   
   
 Avrankou_coluzzii_Delta_3R_42095383 
   
   
   
 Avrankou_coluzzii_Delta_3R_43486612 
   
   
   
 Avrankou_coluzzii_Delta_3R_43532647 
   
   
   
 Avrankou_coluzzii_Delta_X_16349957 
   
   
   
 
 Baguida_ gambiae _Delta 
   
   
  Baguida_gambiae_Delta_2R:3411049  
 Baguida_gambiae_Delta_2R:3453444  
 Baguida_gambiae_Delta_2R:52110329  
 Baguida_gambiae_Delta_2R:53504721  
 Baguida_gambiae_Delta_3R:50086331  
 Baguida_gambiae_Delta_3R:50126025  
 Baguida_gambiae_Delta_2R_3411049 
   
   
   
 Baguida_gambiae_Delta_2R_3453444 
   
   
   
 Baguida_gambiae_Delta_2R_52110329 
   
   
   
 Baguida_gambiae_Delta_2R_53504721 
   
   
   
 Baguida_gambiae_Delta_3R_50086331 
   
   
   
 Baguida_gambiae_Delta_3R_50126025 
   
   
   
 
 Korle-Bu_ coluzzii _Delta 
   
   
  Korle-Bu_coluzzii_Delta_2R:19795384  
 Korle-Bu_coluzzii_Delta_2R:19935256  
 Korle-Bu_coluzzii_Delta_2R:24507343  
 Korle-Bu_coluzzii_Delta_2R:24528127  
 Korle-Bu_coluzzii_Delta_2R:24867722  
 Korle-Bu_coluzzii_Delta_2R:40748811  
 Korle-Bu_coluzzii_Delta_2R:40775991  
 Korle-Bu_coluzzii_Delta_2R:49194797  
 Korle-Bu_coluzzii_Delta_2R:58948322  
 Korle-Bu_coluzzii_Delta_2R:59097397  
 Korle-Bu_coluzzii_Delta_2R:59368196  
 Korle-Bu_coluzzii_Delta_X:15660088  
 Korle-Bu_coluzzii_Delta_X:15703693  
 Korle-Bu_coluzzii_Delta_X:15798349  
 Korle-Bu_coluzzii_Delta_X:15836204  
 Korle-Bu_coluzzii_Delta_X:19194835  
 Korle-Bu_coluzzii_Delta_2R_19795384 
   
   
   
 Korle-Bu_coluzzii_Delta_2R_19935256 
   
   
   
 Korle-Bu_coluzzii_Delta_2R_24507343 
   
   
   
 Korle-Bu_coluzzii_Delta_2R_24528127 
   
   
   
 Korle-Bu_coluzzii_Delta_2R_24867722 
   
   
   
 Korle-Bu_coluzzii_Delta_2R_40748811 
   
   
   
 Korle-Bu_coluzzii_Delta_2R_40775991 
   
   
   
 Korle-Bu_coluzzii_Delta_2R_49194797 
   
   
   
 Korle-Bu_coluzzii_Delta_2R_58948322 
   
   
   
 Korle-Bu_coluzzii_Delta_2R_59097397 
   
   
   
 Korle-Bu_coluzzii_Delta_2R_59368196 
   
   
   
 Korle-Bu_coluzzii_Delta_X_15660088 
   
   
   
 Korle-Bu_coluzzii_Delta_X_15703693 
   
   
   
 Korle-Bu_coluzzii_Delta_X_15798349 
   
   
   
 Korle-Bu_coluzzii_Delta_X_15836204 
   
   
   
 Korle-Bu_coluzzii_Delta_X_19194835 
   
   
   
 
 Madina_ gambiae _Delta 
   
   
  Madina_gambiae_Delta_2L:14079208  
 Madina_gambiae_Delta_2L:14235728  
 Madina_gambiae_Delta_2L:14276791  
 Madina_gambiae_Delta_2L:15136081  
 Madina_gambiae_Delta_2L:15613228  
 Madina_gambiae_Delta_2R:24638486  
 Madina_gambiae_Delta_2R:28466877  
 Madina_gambiae_Delta_3L:10952426  
 Madina_gambiae_Delta_3L:10990152  
 Madina_gambiae_Delta_3L:13952703  
 Madina_gambiae_Delta_3L:14016877  
 Madina_gambiae_Delta_3L:14034923  
 Madina_gambiae_Delta_X:14317604  
 Madina_gambiae_Delta_X:14369513  
 Madina_gambiae_Delta_2L_14079208 
   
   
   
 Madina_gambiae_Delta_2L_14235728 
   
   
   
 Madina_gambiae_Delta_2L_14276791 
   
   
   
 Madina_gambiae_Delta_2L_15136081 
   
   
   
 Madina_gambiae_Delta_2L_15613228 
   
   
   
 Madina_gambiae_Delta_2R_24638486 
   
   
   
 Madina_gambiae_Delta_2R_28466877 
   
   
   
 Madina_gambiae_Delta_3L_10952426 
   
   
   
 Madina_gambiae_Delta_3L_10990152 
   
   
   
 Madina_gambiae_Delta_3L_13952703 
   
   
   
 Madina_gambiae_Delta_3L_14016877 
   
   
   
 Madina_gambiae_Delta_3L_14034923 
   
   
   
 Madina_gambiae_Delta_X_14317604 
   
   
   
 Madina_gambiae_Delta_X_14369513 
   
   
   
 
 Obuasi_ gambiae _Delta 
   
   
  Obuasi_gambiae_Delta_2R:28289136  
 Obuasi_gambiae_Delta_2R:28425075  
 Obuasi_gambiae_Delta_2R:28464858  
 Obuasi_gambiae_Delta_2R:28511074  
 Obuasi_gambiae_Delta_3L:38536401  
 Obuasi_gambiae_Delta_3R:10596787  
 Obuasi_gambiae_Delta_3R:10636722  
 Obuasi_gambiae_Delta_3R:6860221  
 Obuasi_gambiae_Delta_3R:6894288  
 Obuasi_gambiae_Delta_3R:6908903  
 Obuasi_gambiae_Delta_3R:6944074  
 Obuasi_gambiae_Delta_2R_28289136 
   
   
   
 Obuasi_gambiae_Delta_2R_28425075 
   
   
   
 Obuasi_gambiae_Delta_2R_28464858 
   
   
   
 Obuasi_gambiae_Delta_2R_28511074 
   
   
   
 Obuasi_gambiae_Delta_3L_38536401 
   
   
   
 Obuasi_gambiae_Delta_3R_10596787 
   
   
   
 Obuasi_gambiae_Delta_3R_10636722 
   
   
   
 Obuasi_gambiae_Delta_3R_6860221 
   
   
   
 Obuasi_gambiae_Delta_3R_6894288 
   
   
   
 Obuasi_gambiae_Delta_3R_6908903 
   
   
   
 Obuasi_gambiae_Delta_3R_6944074 
   
   
   
 
 Baguida_ gambiae _PM 
   
   
  Baguida_gambiae_PM_X:4481600  
 Baguida_gambiae_PM_X_4481600 
   
   
   
 
 Korle-Bu_ coluzzii _PM 
   
   
  Korle-Bu_coluzzii_PM_2L:20512784  
 Korle-Bu_coluzzii_PM_2L:42166138  
 Korle-Bu_coluzzii_PM_2R:20360304  
 Korle-Bu_coluzzii_PM_2R:38099416  
 Korle-Bu_coluzzii_PM_2R:38131522  
 Korle-Bu_coluzzii_PM_2R:54401839  
 Korle-Bu_coluzzii_PM_3L:12686869  
 Korle-Bu_coluzzii_PM_3R:28478380  
 Korle-Bu_coluzzii_PM_3R:28503198  
 Korle-Bu_coluzzii_PM_3R:28531750  
 Korle-Bu_coluzzii_PM_3R:28562261  
 Korle-Bu_coluzzii_PM_3R:28648828  
 Korle-Bu_coluzzii_PM_2L_20512784 
   
   
   
 Korle-Bu_coluzzii_PM_2L_42166138 
   
   
   
 Korle-Bu_coluzzii_PM_2R_20360304 
   
   
   
 Korle-Bu_coluzzii_PM_2R_38099416 
   
   
   
 Korle-Bu_coluzzii_PM_2R_38131522 
   
   
   
 Korle-Bu_coluzzii_PM_2R_54401839 
   
   
   
 Korle-Bu_coluzzii_PM_3L_12686869 
   
   
   
 Korle-Bu_coluzzii_PM_3R_28478380 
   
   
   
 Korle-Bu_coluzzii_PM_3R_28503198 
   
   
   
 Korle-Bu_coluzzii_PM_3R_28531750 
   
   
   
 Korle-Bu_coluzzii_PM_3R_28562261 
   
   
   
 Korle-Bu_coluzzii_PM_3R_28648828 
   
   
   
 
 Madina_ gambiae _PM 
   
   
  Madina_gambiae_PM_2L:13810050  
 Madina_gambiae_PM_2L:13835438  
 Madina_gambiae_PM_2L:13857697  
 Madina_gambiae_PM_2R:2909619  
 Madina_gambiae_PM_2R:2924284  
 Madina_gambiae_PM_2R:2937977  
 Madina_gambiae_PM_2R:2952746  
 Madina_gambiae_PM_2R:2966523  
 Madina_gambiae_PM_2R:3022442  
 Madina_gambiae_PM_2R:3035126  
 Madina_gambiae_PM_2R:3048496  
 Madina_gambiae_PM_2R:3066659  
 Madina_gambiae_PM_2R:3082612  
 Madina_gambiae_PM_2R:3098810  
 Madina_gambiae_PM_3L:11845057  
 Madina_gambiae_PM_3L:8379569  
 Madina_gambiae_PM_3R:42366567  
 Madina_gambiae_PM_X:10930275  
 Madina_gambiae_PM_X:10958263  
 Madina_gambiae_PM_X:14496426  
 Madina_gambiae_PM_X:14521612  
 Madina_gambiae_PM_2L_13810050 
   
   
   
 Madina_gambiae_PM_2L_13835438 
   
   
   
 Madina_gambiae_PM_2L_13857697 
   
   
   
 Madina_gambiae_PM_2R_2909619 
   
   
   
 Madina_gambiae_PM_2R_2924284 
   
   
   
 Madina_gambiae_PM_2R_2937977 
   
   
   
 Madina_gambiae_PM_2R_2952746 
   
   
   
 Madina_gambiae_PM_2R_2966523 
   
   
   
 Madina_gambiae_PM_2R_3022442 
   
   
   
 Madina_gambiae_PM_2R_3035126 
   
   
   
 Madina_gambiae_PM_2R_3048496 
   
   
   
 Madina_gambiae_PM_2R_3066659 
   
   
   
 Madina_gambiae_PM_2R_3082612 
   
   
   
 Madina_gambiae_PM_2R_3098810 
   
   
   
 Madina_gambiae_PM_3L_11845057 
   
   
   
 Madina_gambiae_PM_3L_8379569 
   
   
   
 Madina_gambiae_PM_3R_42366567 
   
   
   
 Madina_gambiae_PM_X_10930275 
   
   
   
 Madina_gambiae_PM_X_10958263 
   
   
   
 Madina_gambiae_PM_X_14496426 
   
   
   
 Madina_gambiae_PM_X_14521612 
   
   
   
 
 Obuasi_ gambiae _PM 
   
   
  Obuasi_gambiae_PM_2R:3018207  
 Obuasi_gambiae_PM_3L:39831247  
 Obuasi_gambiae_PM_3L:39848513  
 Obuasi_gambiae_PM_3R:48480402  
 Obuasi_gambiae_PM_3R:48535299  
 Obuasi_gambiae_PM_X:14032886  
 Obuasi_gambiae_PM_X:14078096  
 Obuasi_gambiae_PM_X:14375402  
 Obuasi_gambiae_PM_X:14696755  
 Obuasi_gambiae_PM_X:14729619  
 Obuasi_gambiae_PM_2R_3018207 
   
   
   
 Obuasi_gambiae_PM_3L_39831247 
   
   
   
 Obuasi_gambiae_PM_3L_39848513 
   
   
   
 Obuasi_gambiae_PM_3R_48480402 
   
   
   
 Obuasi_gambiae_PM_3R_48535299 
   
   
   
 Obuasi_gambiae_PM_X_14032886 
   
   
   
 Obuasi_gambiae_PM_X_14078096 
   
   
   
 Obuasi_gambiae_PM_X_14375402 
   
   
   
 Obuasi_gambiae_PM_X_14696755 
   
   
   
 Obuasi_gambiae_PM_X_14729619 
   
   
   
 
 
 
