# Supplementary material for: Genome-wide association studies reveal novel loci associated with pyrethroid and organophosphate resistance in Anopheles gambiae s.l
Source: bioRxiv. 2023 Jan 14:2023.01.13.523889. Preprint. [Version 1] doi: 10.1101/2023.01.13.523889 (PMC9882144; doi:10.1101/2023.01.13.523889)
Supplement: Supplement 8 [file media-8.zip › Supplementary_Data_S6.html]

 
 
 
   
   
   
   gwas_summary 
   
   
     
   
 
 
 GAARD GWAS results summary 
 Each plot shows the 1000 most significant SNPs in the GWAS for that
sample set. At the top, the P value for each SNP is shown, colour-coded
by chromosome. Underneath is a heatmap showing the pairwise correlation
between all 1000 SNPs, helping to see groups of SNPs that are in linkage
disequilibrium. Each point in the  P -value plot is lined up with
its column in the heatmap. Beneath the column, lines heatmap columns to
a map of the genome to show the genomic position of each SNP. Black
horizontal bars between the heatmap and the linking lines indicate
genomic regions containing a high density of these top 1000 SNPs (100 kb
windows that contained at least 10 SNPs). 
     
 
  Avrankou_ coluzzii _Delta  
 Baguida_ gambiae _Delta  
 Korle-Bu_ coluzzii _Delta  
 Madina_ gambiae _Delta  
 Obuasi_ gambiae _Delta  
 Baguida_ gambiae _PM  
 Korle-Bu_ coluzzii _PM  
 Madina_ gambiae _PM  
 Obuasi_ gambiae _PM  
 
 Avrankou_ coluzzii _Delta 
   
   
 
 Baguida_ gambiae _Delta 
   
   
 
 Korle-Bu_ coluzzii _Delta 
   
   
 
 Madina_ gambiae _Delta 
   
   
 
 Obuasi_ gambiae _Delta 
   
   
 
 Baguida_ gambiae _PM 
   
   ___ 
 Korle-Bu_ coluzzii _PM 
   
   
 
 Madina_ gambiae _PM 
   
   
 
 Obuasi_ gambiae _PM 
   
   
 
 
 
